# Early and late changes in the normal mouse bladder reservoir function due to irradiation and cis-DDP.

**DOI:** 10.1038/bjc.1992.224

**Published:** 1992-07

**Authors:** F. Lundbeck, J. Overgaard

**Affiliations:** Danish Cancer Society, Department of Experimental Clinical Oncology, Aarhus.

## Abstract

Early and late changes in the reservoir function of the mouse bladder were investigated after radiation alone or a combination of radiation and cisplatinum (cis-DDP). Bladder function was investigated by repeated cystometries. Treatments consisted of either single fraction radiation (5-10-15-25-30 Gy) or 20 Gy in combination with cis-DDP (6 mg kg-1; i.p.) administered at various time intervals from 14 days before until 14 days after radiation. At two selected time intervals (15 min and 4 h before) radiation was given at different dose levels (5-10-15-20 Gy). Within 30 days after irradiation a dose-dependent early response was noticed both in the radiation alone group and the group where cis-DDP was administered 15 min before radiation. The dose-response curve showed a slight but non-significant shift to the left in the combined treatment group (dose effect factor (DEF) = 1.18). Investigation of the early change in bladder reservoir function in the animals treated with 20 Gy alone or a combination of 20 Gy plus cis-DDP at various intervals in relation to irradiation demonstrated a significant increase in response when cis-DDP was administered 24 h and 15 min before and 4 h, 72 h and 336 h after 20 Gy (P less than 0.05). The reversible nature of the early damage was demonstrated. Late response was irreversible and significantly increased in most groups were cis-DDP was administered from 168 h before until 72 h after compared to radiation alone. Comparing groups treated with radiation alone with groups where cis-DDP was administered 15 min and 4 h before radiation revealed DEF values up to 1.45 (P less than 0.05), reflecting the significantly larger response in combined treatment groups. Survival was significantly decreased in all combined treatment groups compared to groups treated with radiation only and likewise survival was decreased in the group treated by cis-DDP alone compared to control (no treatment at all).


*Br. J. Cancer* (1992), **66**, 99–105 © Macmillan Press Ltd., 1992

### Early and late changes in the normal mouse bladder reservoir function due to irradiation and cis-DDP

F. Lundbeck[1,2] & J. Overgaard[1]

[1]*Danish Cancer Society, Department of Experimental Clinical Oncology, Nørrebrogade 44,* [2]*Department of Urology and Institute of Experimental Clinical Research, University of Aarhus, Aarhus Municipal Hospital, DK-8000 Aarhus C, Denmark.*

**Summary** Early and late changes in the reservoir function of the mouse bladder were investigated after radiation alone or a combination of radiation and cisplatinum (cis-DDP). Bladder function was investigated by repeated cystometries. Treatments consisted of either single fraction radiation (5-10-15-25-30 Gy) or 20 Gy in combination with cis-DDP (6 mg $kg^{-1}$; i.p.) administered at various time intervals from 14 days before until 14 days after radiation. At two selected time intervals (15 min and 4 h before) radiation was given at different dose levels (5-10-15-20 Gy). Within 30 days after irradiation a dose-dependent *early response* was noticed both in the radiation alone group and the group where cis-DDP was administered 15 min before radiation. The dose-response curve showed a slight but non-significant shift to the left in the combined treatment group (dose effect factor (DEF) = 1.18). Investigation of the early change in bladder reservoir function in the animals treated with 20 Gy alone or a combination of 20 Gy plus cis-DDP at various intervals in relation to irradiation demonstrated a significant increase in response when cis-DDP was administered 24 h and 15 min before and 4 h, 72 h and 336 h after 20 Gy ($P < 0.05$). The reversible nature of the early damage was demonstrated.

*Late response* was irreversible and significantly increased in most groups were cis-DDP was administered from 168 h before until 72 h after compared to radiation alone. Comparing groups treated with radiation alone with groups where cis-DDP was administered 15 min and 4 h before radiation revealed DEF values up to 1.45 ($P < 0.05$), reflecting the significantly larger response in combined treatment groups. Survival was significantly decreased in all combined treatment groups compared to groups treated with radiation only and likewise survival was decreased in the group treated by cis-DDP alone compared to control (no treatment at all).

Currently, one of the major interests in the treatment of patients with deep muscle-invasive bladder cancer is whether chemotherapy can achieve a prolonged survival when given in combinations with either of the two basic treatment modalities, namely radical cystectomy and curative irradiation. One of the most potent chemotherapeutic agents in urological cancer at present seems to be cis-platinum (cis-diamminedichloroplatinum (II), cis-DDP) which in combinations with methotrexate, vinblastine and doxorubicin has been shown to result in a 30% complete tumour response in patients with advanced disease (Sternberg *et al.*, 1988).

Results from *in vitro* and *in vivo* experimental studies have suggested that cis-DDP exerts a greater than additive effect on tumours when combined with radiation (Overgaard & Kahn, 1981; Lelieveld *et al.*, 1985). However, from a therapeutic point of view, a major aspect in treating malignant tumours is not supra-additivity of tumour cell kill but the achievement of enhanced tumour response without increased normal tissue morbidity (Moore & Mendelsohn, 1972). The combination of cis-DDP and radiation has been investigated *in vivo* in normal tissues. Earlier studies in skin (Overgaard & Kahn, 1981) and intestinal crypt cells (Luk *et al.*, 1979; Von der Maase, 1984) gave somewhat conflicting results, showing an increased effect of cis-DDP combined with irradiation compared with radiation alone in gut (Luk *et al.*, 1979), and no effect in skin studies (Overgaard & Kahn, 1981). More recently the combination was investigated in the mouse (Stewart *et al.*, 1986) and pig (Robbins *et al.*, 1988) kidney, and both studies suggested an additive injury.

To date only two studies have looked at normal tissue damage of the urinary bladder with cis-DDP and irradiation (Lundbeck & Stewart, 1989; Lundbeck *et al.* submitted). Those invesigations, however, were partly directed towards elucidating interstrain differences concerning the early changes in bladder function (Lundbeck & Stewart, 1989), and partly designed to compare two *in vivo* assays using the same endpoints (Lundbeck *et al.*, submitted). Our present study was designed to examine the impact of varying the time interval between administration of single dose irradiation and cis-DDP on early and late damage and survival. In addition, a comparison was made between the effects of either treatment modality alone or in combination, on bladder reservoir function as a function of time after treatment.

### Materials and methods

All experiments were performed using female $C_3D_2F_1$/Bom mice ($C_3$H/Tif♀ × DBA/2♂). The mice were 12–14 weeks old at the start of the experiments and weighed 20–25 g. They were housed six per cage and given a standard laboratory diet and tap water ad libitum.

### *Irradiation and drug treatment*

The irradiation procedure and dosimetry have been described in detail elsewhere (Lundbeck *et al.*, 1989*a*). Basically, mice were anaesthetised (pentobarbital, 60 mg $kg^{-1}$), and the bladder emptied of urine by use of a transurethral catheter. The mouse was then restrained in a plexiglass cylinder snugly fitting into a lead box designed so as to shield the mouse completely except for an oval portal (13 by 8 mm) on each side, thus allowing irradiation of the urinary bladder on two opposing lateral fields. X-rays were generated with a 250 kV constant potential.

Mice given irradiation alone were treated with single doses of either 5, 10, 15, 20, 25 or 30 Gy. In the combined treatment studies animal were irradiated with 20 Gy with the cis-DDP given at various time intervals up to 14 days before and after irradiation. The irradiation dose in these experiments was chosen because an earlier study had shown 20 Gy to be feasible with a consistent response and a tolerable level of toxicity (Lundbeck *et al.*, 1989*a*). Two time intervals were

Correspondence: F. Lundbeck, Danish Cancer Society, Department of Experimental Clinical Oncology, Nörrebrogade 44, DK-8000 Aarhus C, Denmark.
Received 12 September 1991; and in revised form 1 April 1992.

elaborated on further, namely cis-DDP given 15 min and 4 h before irradiation, and the radiation dose levels in these two groups were 5, 10, 15 and 20 Gy.

Any calculation of interval between treatment and response refers to the time of irradiation.

Control groups consisting of no treatment or cis-DDP alone were also included.

Cis-DDP (Platinol, kindly supplied by Bristol Myers, Copenhagen, Denmark) was injected intraperitoneally at a concentration of 0.3 mg $ml^{-1}$. In all experiments a single dose of 6 mg $kg^{-1}$ was given.

### *Assay for bladder reservoir function*

The bladder filling technique has been described previously (Lundbeck *et al.*, 1989*b*). Briefly, the bladder of the anaesthetised mouse was emptied by means of a transurethral catheter. Thereafter the catheter was replaced by a similar, but fluid-filled catheter connected to an infusion pump (0.1 ml $min^{-1}$ isotonic saline), a pressure transducer and an ink jet recorder. When leaking around the catheter occurred, the infusions were stopped. Two days before the start of the experiment a cystometry was performed on each mouse as a control. After treatment the mice were repeatedly investigated on an average of six times during the first 30 days. Thereafter the number of bladder fillings was gradually reduced to one or two cystometries monthly until the late radiation damage was manifest. The control animals were similarly studied until death.

### *Endpoint and evaluation of data*

The median initial volume at an intravesical pressure of 20 mgHg was 246 µl. Response was defined by reduction to less than 50% of that value (123 µl), irrespective of the individual pretreatment volume (Bentzen *et al.*, in press).

In selected groups, i.e. irradiation alone and cis-DDP administered 4 h and 15 min before radiation, dose response curves were performed to evaluate the early and late damage. The curves reflecting early damage were estimated by calculation of the percentage of responding mice from the number of available mice at each radiation dose level within the first 30 days after treatment. Logit analyses were performed allowing an estimation of $RD_{50}$ (the radiation dose inducing a response in 50% of the animals). Similar calculations were performed in the same groups of animals at selected times beyond 30 days to evaluate the late damage.

The combined drug-radiation effect was expressed in terms of the dose effect factor (DEF). This DEF was defined as the ratio between the $RD_{50}$ values for radiation alone and when radiation was combined with cis-DDP. A comparison between the estimated $RD_{50}$ was performed as described recently (Grau *et al.*, 1990).

To compare the response and validate the influence of interval between administration of cis-DDP and irradiation on the early and late response with the changes seen after irradiation alone the percentage of responding mice at each time interval was calculated. Thus the early damage was investigated between days 1 to 30 and the late damage at days 70, 110, 150, 190, 230, 270 and 310 after irradiation. Comparison with the group treated with 20 Gy alone was performed by $\chi^2$-test.

The probability of developing late damage and the probability of survival were both calculated employing the Kaplan-Meier analysis (Kaplan & Meier, 1958), and comparisons were performed using the log rank test (Peto *et al.*, 1977).

### Results

### *Early response*

The effect of varying the time interval between cis-DDP and radiation on the reservior function of the bladder for both early and late damage is illustrated in Figure 1. An increased early response is seen in the interval from 24 h before to 336 h after irradiation, although at 4 h before and 2 min after irradiation the results were the same as seen in the animals irradiated alone. None of the mice treated with cis-DDP ($n = 11$) alone or in the untreated control group responded ($n = 52$).

Figure 2 depicts the dose-response curves for the early response to two selected groups: irradiation alone and cis-DDP administered 15 min before irradiation. The curve for the combination treatment is shifted to the left, reflecting the increased number of responders at various dose levels. At 20 Gy, the difference in numbers of responders is significant ($P < 0.05$), as also illustrated in Figure 1. The $RD_{50}$ value was estimated to be 16.62 Gy for radiation alone compared with 14.10 Gy for cis-DDP and radiation. This resulted in a DEF value of 1.18, which however was non-significant ($P = 0.08$).

The reversible nature of the early damage shown in a previous study (Lundbeck *et al.*, 1989*b*) is also illustrated in Figure 1 (day 1–30 and day 70) by the fact that the response to treatment is decreased. Measurements *at* day 30 showed that none of the animals responded, either in the radiation alone group or in the combined treatment groups (data not shown).

### *Late response*

The dynamic changes in bladder volume over time are illustrated in Figure 1. Already 70 days after treatment there is a slight increase from that seen at day 30 in the number of responders in both the 20 Gy group and 20 Gy plus cis-DDP. Animals treated with cis-DDP either 72 h before or 24 h after irradiation differ significantly from the irradiated group to 20 Gy only. From 110 days after treatment and onwards, there is a steady increase in the overall number of responders. The increasing number of responders in the combined treatment groups is at all times generally higher than in the irradiated group only, indicating a shorter time until damage in the former groups. No animals in the group treated by cis-DDP alone responded, nor did any of the non-treated mice respond during the whole period.

To illustrate the change in isoeffect dose during the observation period, $RD_{50}$ values were estimated in three selected groups, i.e. radiation alone (5–30 Gy) and cis-DDP administered 4 h or 15 min before radiation (5–20 Gy) (Table I). Because of the toxicity in the combined regimen it was not possible to increase the irradiation dose beyond 20 Gy leaving only four dose groups in these experiments as compared to six in the irradiation alone group. The Table illustrates two important features. First, in all three treatment groups the $RD_{50}$ decreases with time implying that time is an important factor in the development of late damage. Second, comparing the $RD_{50}$ values in the irradiation alone group with either of the two other two combined treatment groups reveals a substantial and significant reduction in $RD_{50}$ as illustrated by DEF values of up to 1.45. Comparing the DEF values of the two combined regimens substantiates the increased effect of giving cis-DDP 15 min before irradiation as compared with 4 h before.

Figure 3 shows dose-response curves at day 190, estimated from the number of animals available at this time. Comparing the combined treatment groups with the irradiated groups, the decrease of the $RD_{50}$ is reflected as a significant shift to the left in the curves comprising the combined treatment. Figure 4 visualises the isoeffect dose over time for the three selected groups presented in Table I, emphasising the significantly increased effect in the combined treatment groups and illustrating the maximal effect in the group treated by cis-DDP 15 min before radiation.

Table II is composed of two different Kaplan-Meier estimates, i.e. probability of developing late damage (column a) and the probability of survival (column b), in the groups of animals treated with cis-DDP and irradiation (20 Gy). All the groups in each column have been compared with the group treated with radiation alone (20 Gy). A significant

**Figure 1** Percentage of responding mice (bladder volume $\leqslant 123\,\mu l$) at selected time intervals after treatment. Points represent 20 Gy + cis-DDP 6 mg $kg^{-1}$ at various time intervals in relation to irradiation. The area between the dashed lines gives % response after 20 Gy X-rays alone ($\pm$s.d.). ● = significant difference from 20 Gy X-rays alone ($P < 0.05$). O = no significant difference from 20 Gy X-rays alone.

**Figure 2** Dose response curve for acute damage (day 1–30) in two selected groups of animals: ●--- = cis-DDP 15 min before 5–20 Gy; O— = irradiation alone 5–30 Gy. $RD_{50}$ = 14.10 Gy (10.11–19.67, 95% CI) and 16.62 Gy (14.19–19.46, 95% CI), respectively. DEF = 1.18 (0.98–1.42, 95% CI), $P = 0.08$.

increased probability in developing late damage was seen when cis-DDP was administered in the interval from 168 h before until 72 h after 20 Gy except at three intervals, i.e. 24 h and 15 min before and 2 min after irradiation. Comparing all groups with an increased response revealed no significant difference. The right hand side of Table II shows a significantly shortly survival when cis-DDP was administered in the interval from 72 h before until 24 h after irradiation compared to irradiation alone. There is only one exception, i.e. 2 min after irradiation, which is difficult to account for.

Figure 5 illustrates a comparison of the estimated survival 250 days after treatment in each group treated with cis-DDP plus irradiation (20 Gy) *vs* survival in the group treated with 20 Gy alone. It shows that adding cis-DDP to 20 Gy significantly increases the risk of dying in an interval from 72 h before to 24 h after irradiation implicating an increased toxicity in the combined regime. The only exception is the group treated with cis-DDP 2 min after irradiation. When cis-DDP is administered earlier than 72 h before or beyond 24 h after irradiation there is no statistical difference in the survival compared to the survival after 20 Gy alone.

The probability of survival is demonstrated in Figure 6 in five selected groups. Control animals live significantly longer than animals treated with cis-DDP alone, indicating that cis-DDP exerts an effect on survival. Survival is further

**Table I** $RD_{50}$ for three selected groups at various time intervals after treatment: X-ray (5–30 Gy) alone and cis-DDP (6 mg $kg^{-1}$) administered 4 h and 15 min before radiation (5–20 Gy), respectively

| *Days after treatment* | *$RD_{50}$ X-ray 5–30 Gy S/F* | *No of animals* | *$RD_{50}$ Cis-DDP 4 h before X-ray 5–20/Gy S/F* | *No of animals* | *$DEF_1$* | *$RD_{50}$ Cis-DDP 15 min before X-ray 5–20 Gy* | *No of animals* | *$DEF_2$* | *$DEF_2/DEF_1$* |
|---|---|---|---|---|---|---|---|---|---|
| 70 | 25.27 (21.20–30.12) | 129 | 23.34 (13.48–40.40) | 68 | 1.08 (0.82–1.43) | 19.44 (11.87–31.86) | 53 | 1.30[a] (1.01–1.68) | 1.20 (1.41–3.48) |
| 110 | 23.12 (20.24–26.42) | 125 | 20.45 (14.88–28.11) | 67 | 1.13 (0.95–1.34) | 15.99 (11.64–21.95) | 48 | 1.45[a] (1.22–1.72) | 1.28[a] (1.04–1.57) |
| 150 | 20.44 (18.61–22.46) | 115 | 17.47 (13.94–21.89) | 67 | 1.17[a] (1.04–1.32) | 16.12 (11.55–22.52) | 47 | 1.27[a] (1.07–1.50) | 1.08 (0.90–1.30) |
| 190 | 19.98 (17.52–22.78) | 96 | 16.79 (13.16–21.42) | 59 | 1.19[a] (1.03–1.38) | 14.80 (11.23–19.51) | 44 | 1.35[a] (1.15–1.58) | 1.13 (0.96–1.34) |
| 230 | 17.84 (15.95–19.96) | 72 | 15.70 (12.77–19.30) | 54 | 1.14[a] (1.00–1.28) | 14.84 (11.76–18.73) | 46 | 1.20[a] (1.05–1.37) | 1.06 (0.92–1.22) |
| 270 | 17.43 (15.11–20.09) | 64 | 16.20 (12.37–21.22) | 48 | 1.08 (0.92–1.26) | 13.08 (9.63–17.77) | 36 | 1.33[a] (1.12–1.58) | 1.24[a] (1.03–1.49) |
| 310 | 16.00 (13.87–18.45) | 52 | 15.97 (11.54–22.09) | 43 | 1.00 (0.84–1.20) | 12.32 (4.32–35.13) | 26 | 1.30[a] (1.07–1.57) | 1.30[a] (1.02–1.65) |

DEF = dose effect factor. $DEF_1$ = X-ray *vs* cis-DDP 4 h before radiation. $DEF_2$ = X-ray *vs* cis-DDP 15 min before radiation. $DEF_2/DEF_1$ = The ratio between cis-DDP 15 min before radiation *vs* cis-DDP 4 h before radiation. [a] = statistical significance ($P<0.05$). All numbers in brackets = 95% confidence interval.

significantly decreased in the combined treatment groups, i.e. cis-DDP adminstered 4 h and 15 min before radiation (20 Gy). An especially marked decrease in survival is encountered in the group treated with cis-DDP 15 min before irradiation (20 Gy), reflecting an increased death rate during the early damage.

**Figure 3** Dose-response curves for late damage in three selected groups at day 190. Curves based on % responders from animals alive at day 190. ○— = X-ray (5–30 Gy); ▲--- = cis-DDP 4 h before X-ray (5–20 Gy); ●--- = cis-DDP 15 min before X-ray (5–20 Gy).

**Figure 4** $RD_{50}$ for three selected groups at various time intervals after treatment. For confidence limits and testing for significance, refer to Table I. ○— = X-ray (5–30 Gy); ▲--- = cis-DDP (6 mg $kg^{-1}$) 4 h before X-ray (5–20 Gy); ●--- = cis-DDP (6 mg $kg^{-1}$) 15 min before X-ray (5–20 Gy).

**Table II** The probability of developing late damage after 20 Gy alone compared with 20 Gy combined with cis-DDP (6 mg $kg^{-1}$) at various time intervals. For each comparing group a Kaplan-Meier estimate was calculated and compared by a log rank test (column A). The same procedure has been performed with respect to the probability of survival (column B)

| *20 Gy vs cis-DDP* | *Column A* | *Column B* |
|---|---|---|
| 336 h before 20 Gy | NS | NS |
| 168 h before 20 Gy | $P<0.01$ | NS |
| 72 h before 20 Gy | $P<0.005$ | $P<0.05$ |
| 24 h before 20 Gy | NS | $P<0.005$ |
| 4 h before 20 Gy | $P<0.025$ | $P<0.025$ |
| 15 min before 20 Gy | NS | $P<0.005$ |
| 2 min after 20 Gy | NS | NS |
| 4 h after 20 Gy | $P<0.01$ | $P<0.005$ |
| 24 h after 20 Gy | $P<0.005$ | $P<0.01$ |
| 72 h after 20 Gy | $P<0.025$ | NS |
| 168 h after 20 Gy | NS | NS |
| 336 h after 20 Gy | NS | NS |

NS = no significant difference.

**Figure 5** A comparison between the estimated survival 250 days after treatment (Kaplan-Meier, log rank). Points represent 20 Gy S/F + cis-DDP 6 mg $kg^{-1}$ at various time intervals in relation to irradiation. The area between dashed lines: 20 Gy S/F ± s.d., ● = significant difference ($P<0.05$) from 20 Gy group, ○ = no significant difference. Bars indicate ± s.d.

In the treatment group where cis-DDP was administered 15 min before irradiation (5–20 Gy) the probability of survival has been further investigated (Figure 7). From this figure it can be seen that the survival decreases significantly with increasing radiation dose, indicating that radiation dose is a crucial element in survival ($P<0.05$).

**Figure 6** Estimated survival curves in five selected groups, $P<0.05$ (Kaplan-Meier, log rank). Cis-DDP dose 6 mg $kg^{-1}$.

**Figure 7** Estimated survival curves in four selected groups, $P<0.05$ (Kaplan Meier, log rank). Cis-DDP dose 6 mg $kg^{-1}$.

### Discussion

The present investigation employs cystometry to evaluate a functional endpoint. A 50% decrease in the median urinary bladder pretreatment volume was used as the endpoint to illustrate the early and long term normal tissue changes after radiation alone compared to the radiation response when combined with cis-DDP at various time intervals.

*Early response*

Our study on the effect of varying the interval between cis-DDP and irradiation indicates an increased response in the combined treatment groups compared to radiation alone in a rather wide time interval, i.e., administering cis-DDP from 24 h before until 336 h after radiation (Figure 1), with some few exceptions. This time interval is somewhat different from other reports (Von der Maase, 1986; Pearson & Steel, 1984) where an increased response was produced only at tighter intervals between cis-DDP and irradiation. Pearson & Steel (1984) performed a time-dependency study on lethality in mice treated with cis-DDP administered at various times before and after irradiation, and found an increased response within the first 30 days after irradiation when the drug was administered 1–2 days before until approximately 7 days after irradiation. Von der Maase (1986), investigating intestinal crypt cells, reported an increased response at a still tighter interval between drug and irradiation, i.e. approximately 24 h before until simultaneous application of the two treatments. Luk *et al.* (1979) demonstrated an increased response in intestinal crypt cells only when the interval was further reduced, i.e. a few hours before until simultaneous treatment. The last study contrasts with our findings, since we could not demonstrate any increased effect when cis-DDP was administered either 4 h before or 2 min after irradiation. Our findings at these two particular intervals are surprising and hard to account for.

In spite of the differences with respect to the interval between cis-DDP and irradiations the previous studies all demonstrate an increased early effect that can be ascribed to some effect from cis-DDP (Luk *et al.*, 1979; Von der Maase, 1986; Pearson & Steel, 1984). Other studies, however, on similar relatively rapidly proliferating normal tissues such as mouse lip mucosa (Landuyt *et al.*, 1986) and mouse skin (Bartelink *et al.*, 1986; Fu & Lam, 1991) have failed to demonstrate any effect due to cis-DDP when administered with radiation, irrespective of the interval between the treatments. Some of these discrepancies may be due to differences in animal strain, radiation doses or scoring methods. But one should also take into consideration the basic differences in the selected endpoints. Our endpoint is a purely functional one, and comparing results deduced from such a non-clonogenic assay with results originating from different clonal assays does imply some difficulties (Michalowski *et al.*, 1984). This issue has been amply illustrated in a recent publication comparing the functional glucose absorption and the clonogenic damage evaluated as cell survival in crypt cells in the same mice. Although the results from both endpoints showed a decrease as a function of radiation dose there was no quantitative correlation between the two (Overgaard & Matsui, 1990).

In a previous bladder study we demonstrated a clear dose-response relationship in the early response to irradiation alone (Lundbeck *et al.*, 1989a). A similar dose dependency was found in the present investigation (Figure 2) when cis-DDP was administered 15 min before radiation. The curve is shifted somewhat to the left compared to the radiation only curve, making the immediate suggestion of some effect of cis-DDP. The DEF value was calculated to 1.18, which, however, is not statistically significant. This may seem somewhat contradictory to the finding in Figure 1, where a significant difference was demonstrated at 20 Gy. However, the difference in data analysis in the two figures should be considered. The curves are rather steep, which means that a significant difference between $RD_{50}$ values will be more difficult to detect compared with a difference in response. Furthermore, the wide confidence limits, reflecting an uneven distribution of responders among the radiation dose levels in the combined treatment group, probably accounts for the lack of statistical difference.

The exact mechanism for the early and transient damage in the bladder is not fully understood either for radiation alone, or for radiation combined with cis-DDP. Cell kinetic investigations (Stewart *et al.*, 1980) after radiation alone have shown an exceptionally slow turnover in the normal urothelium, and no change has been noticed in proliferation until the late damage was about to be manifest. So the early damage leading to an hyperactive and irritated bladder is not supposed to be a result of cell death and sloughing of the urothelium, as experienced after administering cyclophosphamide (Lundbeck & Stewart, 1989; Stewart, 1985). One might then assume some changes in the contractile mechanisms in the bladder, reflected in the decreased compliance. However, in a recent pharmacological *in vitro* study of nerve and muscular functions in the mouse bladder wall (Lundbeck & Sjögren, 1991) it was not possible to indicate any differences between irradiated (25 Gy) and non-treated mouse bladders concerning the release mechanism of acetylcholine, cholinergic or non-cholinergic nerve activation, prostaglandin function or potassium channel activation.

cis-DDP by itself did not produce a change in the bladder reservior function, but the present study clearly demonstrates some interaction between the drug and radiation when combined. Since the early response takes place largely when the drug is administered *after* radiotherapy, the effect does not seem to be a true sensitisation.

*Late response*

Although the early change in bladder capacity does pose some clinical problems the degree of late damage on normal tissue is the all important issue in the treatment of malignant tumours with radiotherapy either alone or in combination with cytostatic drugs.

An earlier study (Lundbeck *et al.*, 1989*a*) demonstrated a late response to different radiation doses when given alone. The isoeffect curve indicated that over time an increasing number of animals did respond in lower dose groups reflected by a decrease in the $RD_{50}$ (Lundbeck *et al.*, 1989*a*). When combining cis-DDP either 15 min or 4 h before irradiation the same feature is illustrated in the present study (Table I, Figure 4) and furthermore the $RD_{50}$ is found to be significantly lower in both the combined treatment groups compared to radiation alone. The decrease in isoeffect dose – reflected by DEF values up to 1.45 – is approximately 30% in the group treated with cis-DDP 15 min before irradiation, thus indicating this interval to be especially damaging. This finding is admittedly somewhat contradictory to the observation in Figure 1, where the difference in response between the group treated with cis-DDP 15 min before 20 Gy and the irradiation only group (20 Gy) is not statistically significant except 110 days after treatment. This inconsistency between Figure 1 and 4 on this point can, however, be ascribed to the fairly small number of animals available at this particular dose level. Estimation of a dose response curve, on the other hand, comprising several dose points decreases the risk of a type 2 error considerably, provided that the confidence limit is reasonably tight.

The time course for the changes in the bladder reservoir function following the administration of cis-DDP either before or after irradiation (Figure 1 and Table II) suggested that the late response took place at an earlier time in the combined treatment groups as compared to the radiation alone group. This may be a true observation, but may also reflect a more pronounced damage to the compliance capacity of the bladder wall, therefore resulting in an earlier detection of the damage. The distinction between these two possibilities is, however, not possible from the present analysis, since this would imply a change in endpoint definition. At present, this is qualitative rather than quantitative. Similarly, estimation of the probability of developing late damage (Table II) revealed a significantly shortened interval from treatment until response or a significantly increased response at the same time interval (after treatment) in all but three groups treated with cis-DDP from 168 h before until 72 h after irradiation (20 Gy) compared with irradiation alone (20 Gy). The three intervals were 24 h and 15 min before and 2 min after irradiation.

While the non-significant finding concerning the interval 15 min before irradiation has been discussed above, it is hard to account for the similar finding in Table II concerning 24 h before and 2 min after irradiation, except for the fact that there was a signfciant increase in mortality in the animals treated with cis-DDP 24 h before irradiation compared with irradiation alone evaluated by a log rank test. On the other hand, the percentage of responding animals during the whole observation period is very low (Figure 1) so the lack of significant difference is probably true and may well reflect experimental variability. The present results do not enable this issue to be resolved any further. Although the results are somewhat variable, they nevertheless indicate a general increase in the effect when cis-DDP is combined with radiation on the late responding bladder tissue as long as the interval between drug and radiation does not exceed 1 week before until 72 h after.

Another late responding tissue, the mouse kidney, has also been studied extensively to evaluate the combined effect of cis-DDP and radiation. This study (Stewart *et al.*, 1986) demonstrated a DEF value of approximately 1.3 reflecting a significant renal toxicity when cis-DDP was combined with radiation, administered either before or within 2 weeks after irradiation. In contrast to the kidney studies and our present investigation, Dewit *et al.* (1987), concluded from his time course investigations on stricture formation in mouse rectum that there was no effect of cis-DDP, when combined with radiation. Those studies on late damage even demonstrated an increase in $RD_{50}$ when cis-DDP was combined with irradiation compared with irradiation alone.

Although cis-DDP alone did not appear to change the bladder volume either acutely or chronically, the drug itself did have an influence on the survival of the animals, those treated with cis-DDP alone having a significantly shorter survival than controls. The difference in survival in these two groups may be somewhat difficult to account for, since the applied dose (6 mg $kg^{-1}$) is not supposed to exert a late affect on the kidney function. In a recent study by Stewart *et al.* (1986) it was shown that the early damage inflicted on the kidney function after administering 6 mg $kg^{-1}$ cis-DDP subsides, and the kidney function – evaluated by the $^{51}$Cr-EDTA clearance – returned to normal after approximately 10 weeks. Thereafter the clearance remained almost normal. In animals treated with higher drug doses, on the other hand, a dose dependent increase in the amount of renal damage persists, with no recovery for up to 40 weeks. Cis-DDP does, however, also exert some haematological toxicity resulting in anaemia or internal haemorrhage (Robbins *et al.*, 1988; Siddik *et al.*, 1987). However, while we cannot exclude anaemia, we have not encountered haemorrhage.

In a previous study (Lundbeck *et al.*, 1989*a*) we have discussed survival in relation to a range of radiation dose levels (5–30 Gy), and a clear dose-dependency was demonstrated. The same feature was encountered in the groups treated with cis-DDP 15 min before 5–20 Gy (Figure 7), suggesting that radiation dose is the most important factor. Comparing equal radiation dose levels with and without the drug administered at this interval by means of Kaplan-Meier plots indicated that the radiation dose had to be of a certain magnitude before an increased mortality in the combined treatment groups could be demonstrated (data not shown). Thus, not until a dose level of 20 Gy did a significant increased mortality appear in the combined treatment groups *vs* the 20 Gy alone group. The reason for this may be the general ability of tissues to regenerate or otherwise compensate for trauma up to a certain level. This issue has been discussed recently by Hendry (Hendry & Thames, 1986) with special reference to radiation damage.

Combining cis-DDP with 20 Gy radiation at time intervals of 72 h before to 24 h after irradiation resulted in a significantly increased mortality (Figure 5, Table II). There seems only to be one exception and that is 2 min after irradiation, which is hard to account for. The groups that produce the increased mortality seem to correlate well with those showing an increased response compared to radiation alone (Figure 5, Figure 6, Table I), but this assumption does not imply that non-responders in the same groups live longer. Our data analyses, however, do not at present enable us to elaborate further on this pertinent issue.

During the first weeks after cis-DDP and irradiation to 20 Gy, a great number of animals lost 10–20% of their body weight, resembling the animals with those in previous experiments treated up to 25 and 30 Gy. The main problem for the animal during that period is probably imbalance of water and electrolytes partly due to irradiation damage to the small intestines in spite of shielding (Lundbeck *et al.*, 1989*a*) and partly in the cis-DDP treated animals due to kidney damage. No animal was deliberately killed during the early phase, except for animals that appeared to be suffering, the major concern being to allow the animal to exhibit late bladder damage. Even after having developed the late damage most of the animals were left alive to accomplish a true survival. However, animals at a late state developing gross tumours were killed, although this was less than 5%. Animals dying spontaneously were always found in a state where postmortems would be meaningless. When postmortems were performed, no obvious reason for dying could be demonstrated such as internal haemorrhage, as found by others (Robbins *et al.*, 1988; Siddik *et al.*, 1987). Neither have we encountered convincing evidence of animals dying from stenosis of the ureters resulting in large hydronephrosis as described by others (Knowles & Trott, 1987). The urine was routinely checked by stix when the bladder was emptied during the experiments, but no sign of bacteriurea has been found. Infections resulting in abscess formation have however not been noticed.

We believe that differences in sensitivity is one of the main reasons for the variations in mortality encountered in different experiments and laboratories. The issue of sensitivity has been discussed in two previous papers (Lundbeck & Stewart, 1989; Lundbeck *et al.*, 1989*a*), one of these (Lundbeck & Stewart, 1989) especially focusing on the evident strain differences in respect to sensitivity towards different treatments, involving irradiation, cyclophosphamide and cis-DDP. Differences in mortality due to difference in sensitivity to cis-DDP has been reported in other animals (Robbins *et al.*, 1988).

In conclusion the present investigation shows an early reversible followed by an irreversible late damage to the reservoir function of the mouse bladder, both after irradiation alone and combined with cis-DDP. The early damage is most severe when cis-DDP is administered from 24 h before until 336 h after radiation with some few exceptions; late damage is most prominent in the combined treatment groups when cis-DDP is applied at a fairly short interval in relation to radiation (not exceeding 1 week). Survival is decreased considerably when cis-DDP is combined with radiation compared to radiation alone, but although cis-DDP *per se* significantly decreases survival compared to control, radiation dose is the all important factor for survival.

We thank Michael R. Horsman, Ph.D., for fruitful discussions and careful assistance in correcting our English.

### References

BARTELINK, H., KALLMAN, R.F., RAPACHHIETTA, D. & HART, G.A.M. (1986). Therapeutic enhancement in mice by clinically relevant dose and fractionation schedules of cis-diamminedichloroplatinum (II) and irradiation. *Radiother. Oncol.*, **6**, 61–74.

BENTZEN, S.M., LUNDBECK, F., LOFT CHRISTENSEN, L. & OVERGAARD, J. (1992). Fractionation sensitivity and latency of late radiation injury to the mouse urinary bladder. *Radiother. Oncol.* (in press).

DEWIT, L., OUSSOREN, Y. & BARTELINK, H. (1987). Early and late damage in the mouse rectum after irradiation and Cis-diamminedichloroplatinum (II). *Radiother. Oncol.*, **8**, 57–69.

FU, K.K. & LAM, K.N. (1991). Early and late effects of cisplatin and radiation at acute and low dose rates on the mouse skin and soft tissues of the leg. *Int. J. Radiat. Oncol. Biol. Phys.*, **20**, 327–332.

GRAU, C., BENTZEN, S.M. & OVERGAARD, J. (1990). Cytotoxic effect of misonidazole and cyclophosphamide on aerobic and hypoxic cells in a C3H mammary carcinoma *in vivo*. *Br. J. Cancer*, **61**, 61–64.

HENDRY, J.H. & THAMES, H.D. (1986). The tissue rescue unit. (Letter to the Editor). *Br. J. Radiol.*, **59**, 628.

KAPLAN, E.L. & MEIER, P. (1958). Nonparametric estimation from incomplete observations. *J. Am. Statist. Ass.*, **53**, 457–481.

KNOWLES, J.F. & TROTT, K.R. (1987). Experimental irradiation of the rat ureter: The effects of field size and the presence of contrast medium on incidence and latency of hydronephrosis. *Radiother. Oncol.*, **10**, 59–66.

LANDUYT, W., ANG, K.K. & VAN DER SCHUEREN, E. (1986). Combinations of single doses and fractionated treatments of cis-dichlorodiammineplatinum (II) and irradiation: effects on mouse lip mucosa. *Br. J. Cancer*, **54**, 579–586.

LELIEVELD, P., SCOLES, M.A., BROWN, J.M. & KALLMAN, R.F. (1985). The effect of treatment in fractionated schedules with combination of X-irradiation and six cytostatic drugs on RIF-1 tumor and normal mouse skin. *Int. J. Radiat. Biol. Phys.*, **11**, 111–121.

LUK, K.H., ROSS, G.Y., PHILLIPS, T.L. & GOLDSTEIN, L.S. (1979). The interaction of radiation and cis-diamminedichloroplatinum (II) in intestinal crypt cells. *Int. J. Radiat. Oncol. Biol. Phys.*, **5**, 1417–1420.

LUNDBECK, F., DJURHUUS, J.CHR. & VAETH, M. (1989*b*). Bladder filling in mice: an experimental in vivo model to evaluate the reservior function of the urinary bladder in a long term study. *J. Urol.*, **141**, 1245–1249.

LUNDBECK, F., OUSSOREN, Y. & STEWART, A.F. (1992). Early and late damage in the mouse bladder due to radiation combined with cyclophosphamide or cis-DDP, evaluated by two different functional assays. *Br. J. Urol.* (submitted).

LUNDBECK, F. & SJÖGREN, C. (1991). A pharmacologic in vitro study of the mouse urinary bladder at the time of the acute damage after irradiation. *J. Urol.* (in press).

LUNDBECK, F. & STEWART, F.A. (1989). Acute changes in the bladder reservoiur function after irradiation alone or in combination with chemotherapy: a matter of mouse strain. *Scand. J. Urol. Nephrol. Suppl.*, **125**, 141–148.

LUNDBECK, F., ULSØ, N. & OVERGAARD, J. (1989*a*). Cystometric evaluation of early and late irradiation damage to the mouse urinary bladder. *Radiother. & Oncol.*, **15**, 383–392.

MICHALOWSKI, A., WHELDON, T.E. & KIRK, J. (1984). Can cell survival parameters be deduced from non-clonogenic assays of radiation damage to normal tissues? *Br. J. Cancer*, **49**, Suppl. VI, 257–261.

MOORE, D.H. & MENDELSOHN, M.L. (1972). Optimal treatment levels in cancer therpay. *Cancer*, **30**, 97–106.

OVERGAARD, J. & KAHN, A.R. (1981). Selective enhancement of radiation response in a C3H mammary carcinoma by cisplatin. *Cancer Treat. Rep.*, **65**, 501–503.

OVERGAARD, J. & MATSUI, M. (1990). Effect of radiation on glucose absorption in the mouse jejunum *in vivo*. *Radiother. Oncol.*, **18**, 71–77.

PEARSON, A.E. & STEEL, G.G. (1984). Chemotherapy in combination with pelvic irradiation: a time dependence study in mice. *Radiother. Oncol.*, **2**, 49–55.

PETO, R., ARMITAGE, P., BRESLOW, N.E. & 6 others (1977). Design and analysis of randomized clinical trials requiring prolonged observation of each patient. II. Analysis and examples. *Br. J. Cancer*, **35**, 1–39.

ROBBINS, M.E.C., ROBINSON, M., REZVANI, M., GOLDINGS, S.J. & HOPEWELL, J.W. (1988). The response of the pig kidney to the combined effects of cisplatin and unilateral renal irradiation. *Radiother. Oncol.*, **11**, 271–278.

SIDDIK, Z.H., BOXALL, F.E. & HARRAP, K.R. (1987). Haematological toxicity of carboplatin in rats. *Br. J. Cancer*, **55**, 375–379.

STERNBERG, C.N., YAGODA, A., HERR, H.W., SHER, H.I., WATSON, R.C., HERR, H.W., MORSE, M.J., SOGANI, P.C, VAUGHAN, E.D., BANDER, N. Jr, WEISELBERG, L.R., GELLER, N., HOLLANDER, P.S., LIPPERMAN, R., FAIR, W.R. & WHITMORE, W.F. (1988). M-VAC (Methotrexate, Vinblastine, Doxorubicin and Cisplatin) for advanced transitional cell carcinoma of the urothelium. *J. Urol.*, **139**, 461–469.

STEWART, A.F. (1985). The proliferative and functional response of mouse bladder to treatment with radiation and cyclophosphamide. *Radiother. Oncol.*, **4**, 353–362.

STEWART, F.A., BOHLKEN, S., BEGG, A. & BARTELINK, H. (1986). Renal damage in mice after treatment with cis-platinum alone or in combination with radiation. *Int. J. Radiat. Oncol. Biol. Phys.*, **12**, 927–933.

STEWART, F.A., DENEKAMP, J. & HIRST, D.G. (1980). Proliferation kinetics of the mouse bladder after irradition. *Cell Tissue Kinet.*, **13**, 75–89.

STEWART, F.A., LUNDBECK, F., OUSSOREN, Y. & LUTS, A. (1991). Acute and late radiation damage in mouse bladder. A comparison of urination frequency and cystometry. *Int. J. Radiol. Oncol. Biol. Phys.*, **21**, 1211–1219.

STEWART, A.F., MICHAEL, B.D. & DENEKAMP, J. (1978). Late radiation damage in the mouse bladder as measured by increased urination frequency. *Radiation Res.*, **75**, 649–659.

VON DER MAASE, H. (1984). Interactions of radiation and adriamycin, bleomycin, mitomycin C or cis-diamminedichloroplatinum (II) in intestinal crypt cells. *Br. J. Cancer*, **49**, 779–786.

VON DER MAASE, H. (1986). Experimental studies on interactions of radiation and cancer chemotherapeutic drugs in normal tissues and solid tumors. *Radiother. Oncol.*, **7**, 47–68.


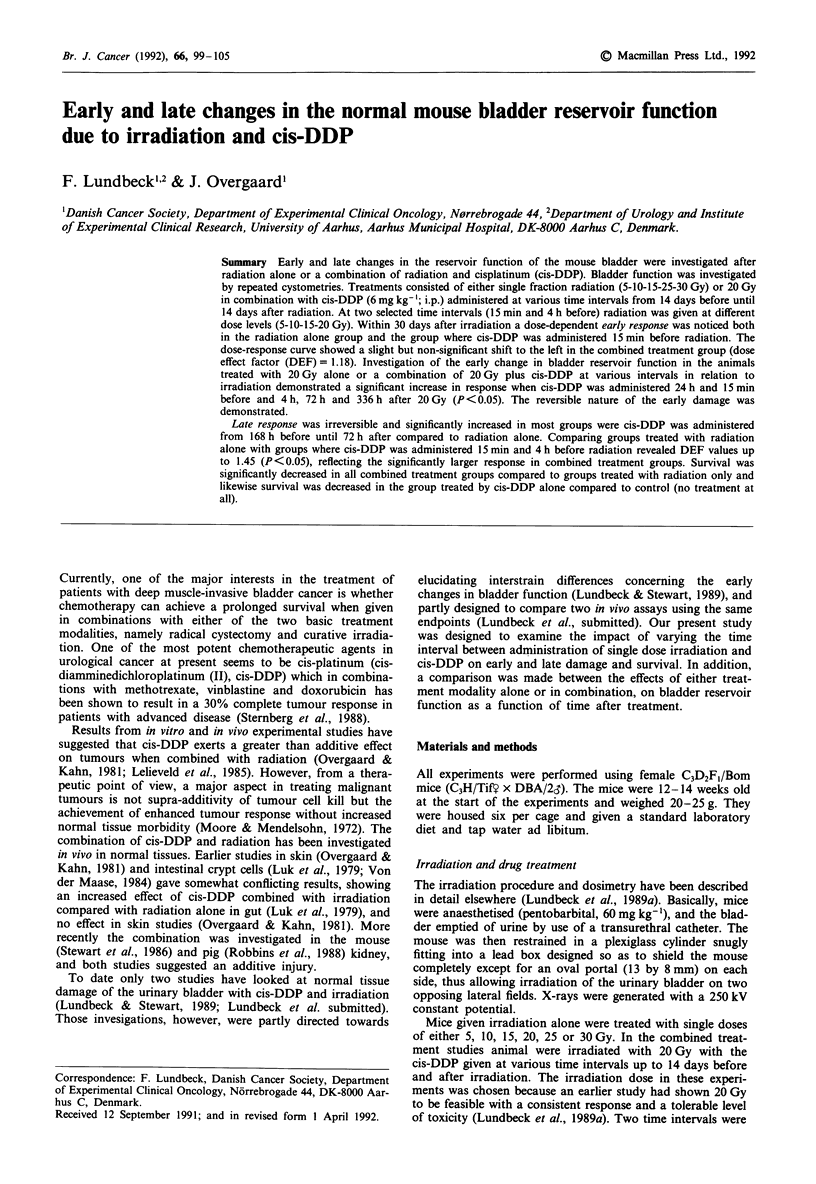

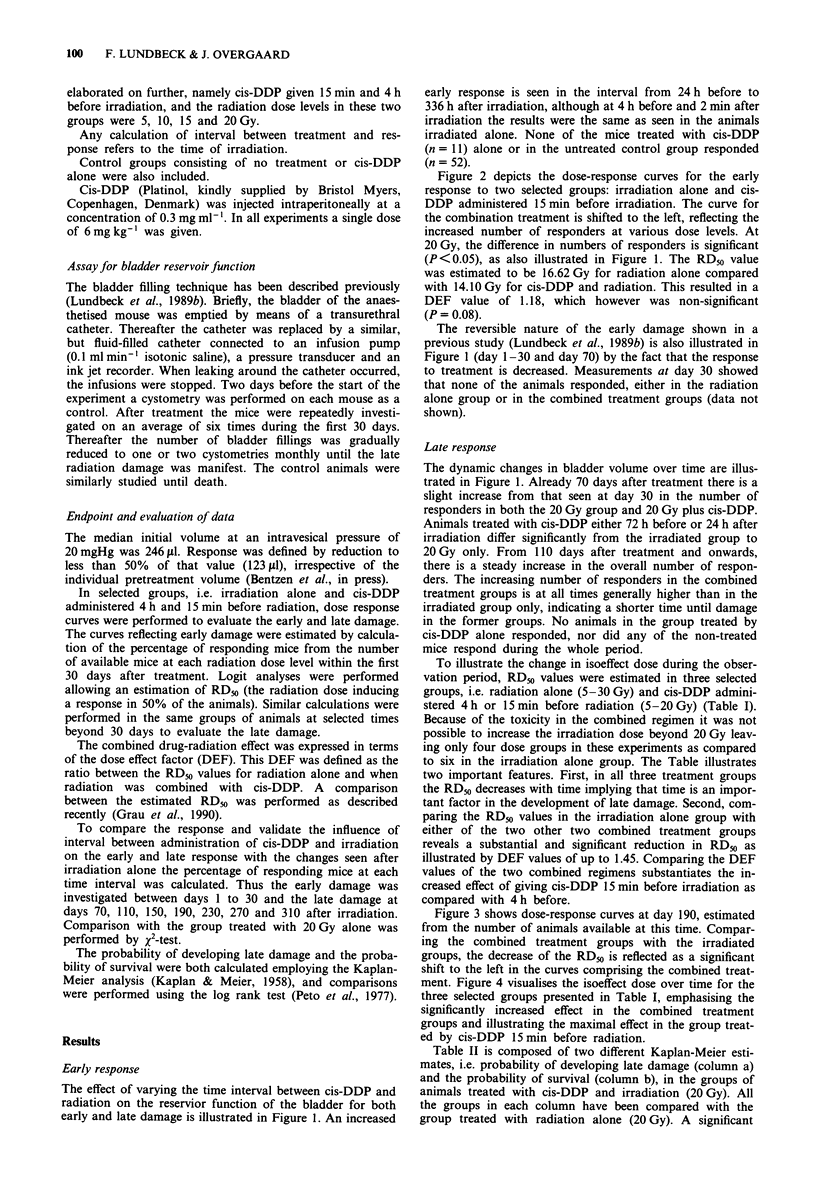

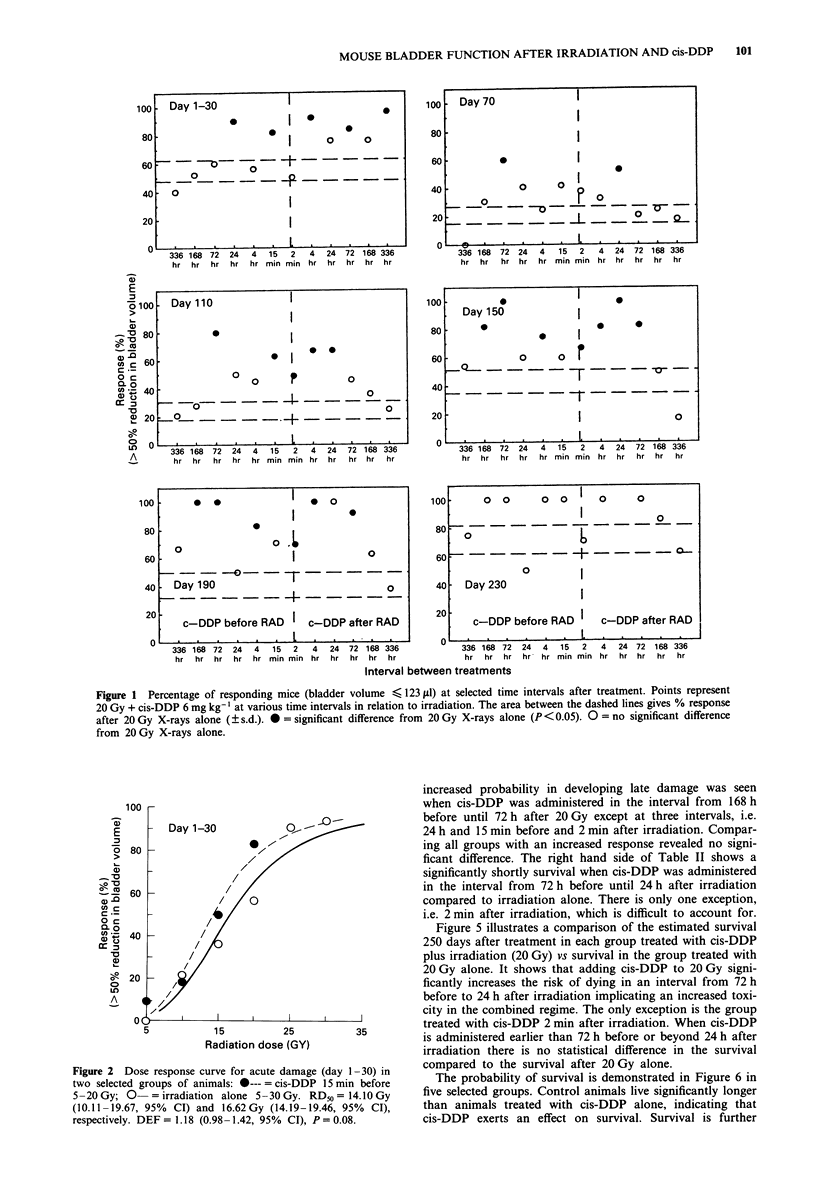

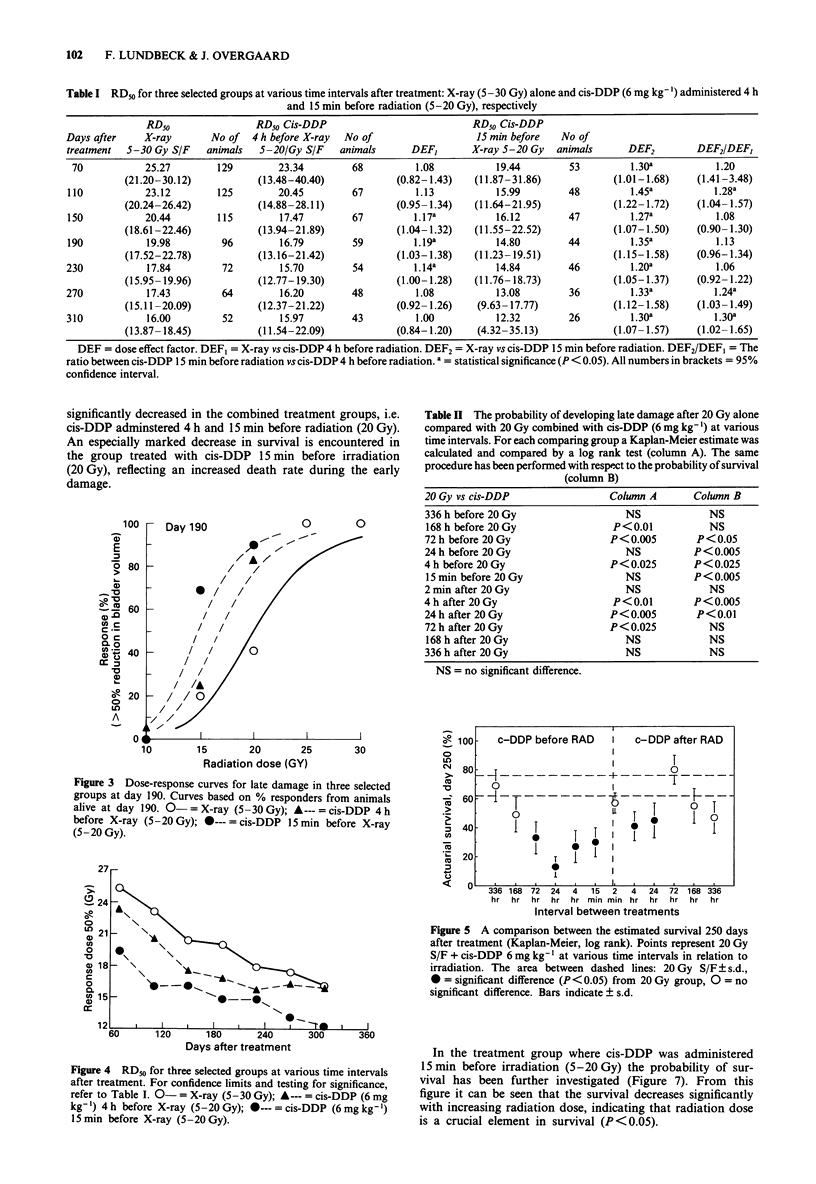

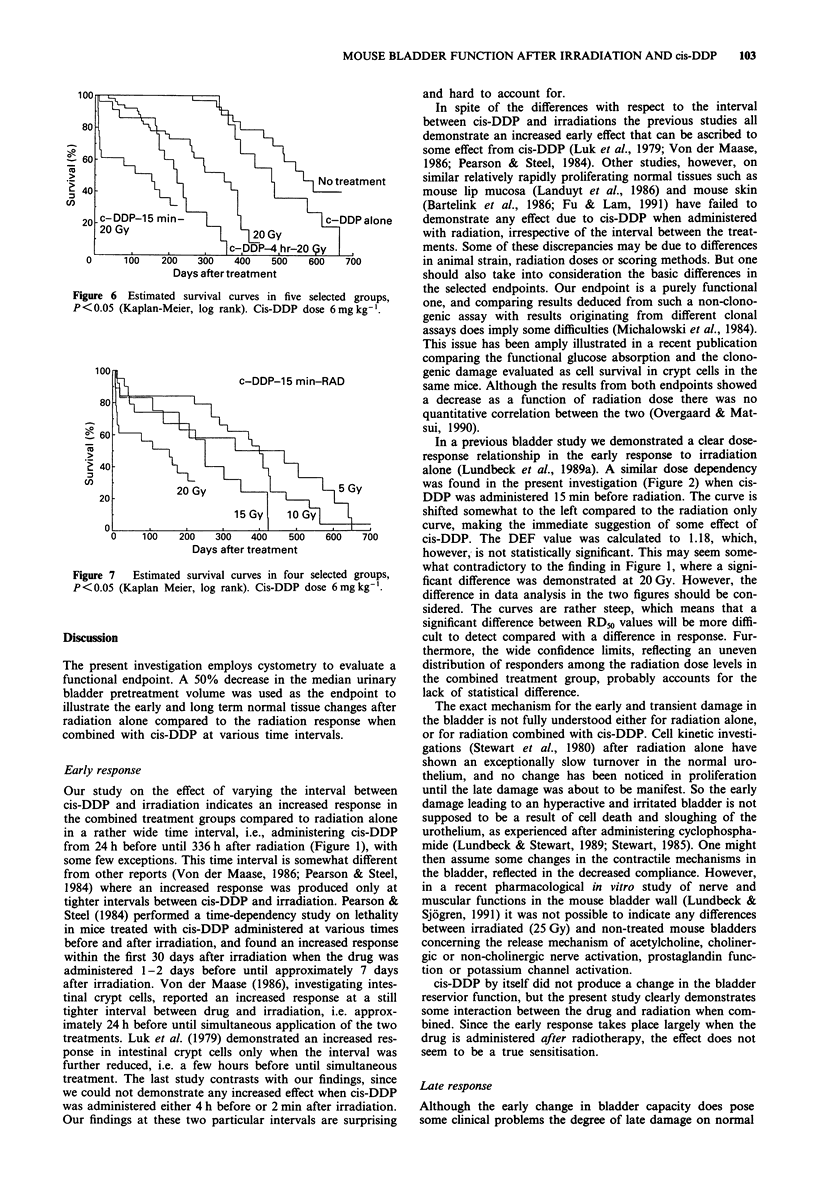

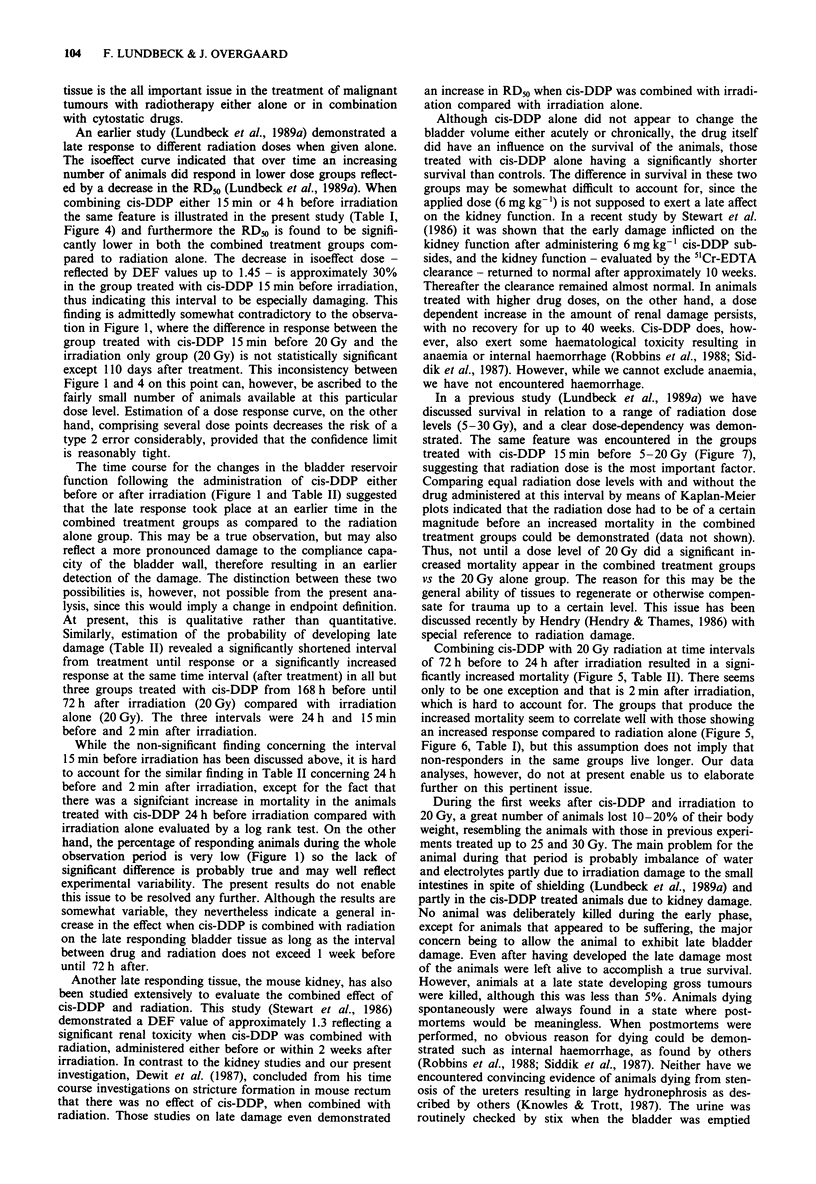

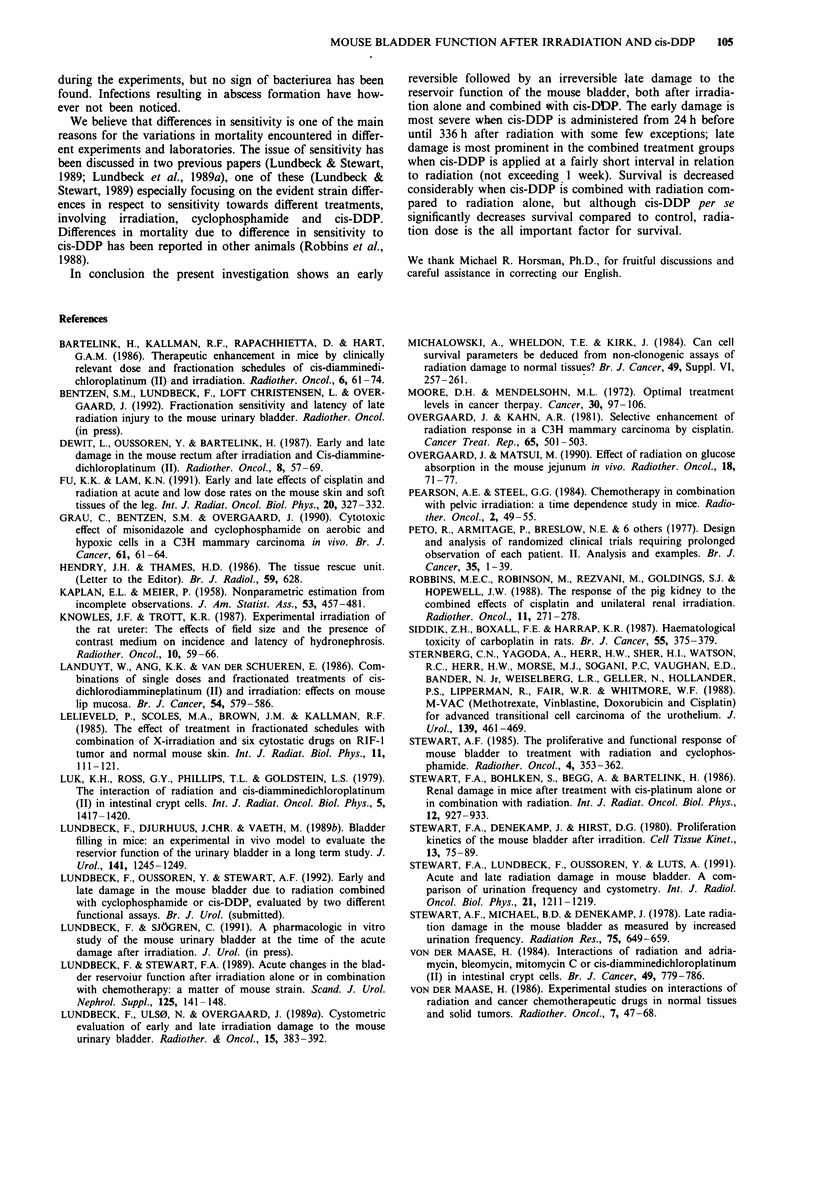


## References

[OCR_01181] Bartelink H., Kallman R. F., Rapacchietta D., Hart G. A. (1986). Therapeutic enhancement in mice by clinically relevant dose and fractionation schedules of cis-diamminedichloroplatinum (II) and irradiation.. Radiother Oncol.

[OCR_01192] Dewit L., Oussoren Y., Bartelink H. (1987). Early and late damage in the mouse rectum after irradiation and cis-diamminedichloroplatinum(II).. Radiother Oncol.

[OCR_01197] Fu K. K., Lam K. N. (1991). Early and late effects of cisplatin and radiation at acute and low dose rates on the mouse skin and soft tissues of the leg.. Int J Radiat Oncol Biol Phys.

[OCR_01201] Grau C., Bentzen S. M., Overgaard J. (1990). Cytotoxic effect of misonidazole and cyclophosphamide on aerobic and hypoxic cells in a C3H mammary carcinoma in vivo.. Br J Cancer.

[OCR_01207] Hendry J. H., Thames H. D. (1986). The tissue-rescuing unit.. Br J Radiol.

[OCR_01215] Knowles J. F., Trott K. R. (1987). Experimental irradiation of the rat ureter: the effects of field size and the presence of contrast medium on incidence and latency of hydronephrosis.. Radiother Oncol.

[OCR_01221] Landuyt W., Ang K. K., van der Schueren E. (1986). Combinations of single doses and fractionated treatments of cis-dichlorodiammineplatinum (II) and irradiation: effect on mouse lip mucosa.. Br J Cancer.

[OCR_01227] Lelieveld P., Scoles M. A., Brown J. M., Kallman R. F. (1985). The effect of treatment in fractionated schedules with the combination of X-irradiation and six cytotoxic drugs on the RIF-1 tumor and normal mouse skin.. Int J Radiat Oncol Biol Phys.

[OCR_01234] Luk K. H., Ross G. Y., Phillips T. L., Goldstein L. S. (1979). The interaction of radiation and cis-diamminedichloroplatinum (II) in intestinal crypt cells.. Int J Radiat Oncol Biol Phys.

[OCR_01242] Lundbeck F., Djurhuus J. C., Vaeth M. (1989). Bladder filling in mice: an experimental in vivo model to evaluate the reservoir function of the urinary bladder in a long term study.. J Urol.

[OCR_01257] Lundbeck F., Stewart F. A. (1989). Acute changes in the bladder reservoir function after irradiation alone or in combination with chemotherapy: a matter of mouse strain.. Scand J Urol Nephrol Suppl.

[OCR_01263] Lundbeck F., Ulsø N., Overgaard J. (1989). Cystometric evaluation of early and late irradiation damage to the mouse urinary bladder.. Radiother Oncol.

[OCR_01268] Michalowski A., Wheldon T. E., Kirk J. (1984). Can cell survival parameters be deduced from non clonogenic assays of radiation damage to normal tissues?. Br J Cancer Suppl.

[OCR_01274] Moore D. H., Mendelsohn M. L. (1972). Optimal treatment levels in cancer therapy.. Cancer.

[OCR_01278] Overgaard J., Khan A. R. (1981). Selective enhancement of radiation response in a C3H mammary carcinoma by cisplatin.. Cancer Treat Rep.

[OCR_01283] Overgaard J., Matsui M. (1990). Effect of radiation on glucose absorption in the mouse jejunum in vivo.. Radiother Oncol.

[OCR_01288] Pearson A. E., Steel G. G. (1984). Chemotherapy in combination with pelvic irradiation: a time-dependence study in mice.. Radiother Oncol.

[OCR_01293] Peto R., Pike M. C., Armitage P., Breslow N. E., Cox D. R., Howard S. V., Mantel N., McPherson K., Peto J., Smith P. G. (1977). Design and analysis of randomized clinical trials requiring prolonged observation of each patient. II. analysis and examples.. Br J Cancer.

[OCR_01299] Robbins M. E., Robinson M., Rezvani M., Golding S. J., Hopewell J. W. (1988). The response of the pig kidney to the combined effects of cisplatin and unilateral renal irradiation.. Radiother Oncol.

[OCR_01305] Siddik Z. H., Boxall F. E., Harrap K. R. (1987). Haematological toxicity of carboplatin in rats.. Br J Cancer.

[OCR_01309] Sternberg C. N., Yagoda A., Scher H. I., Watson R. C., Herr H. W., Morse M. J., Sogani P. C., Vaughan E. D., Bander N., Weiselberg L. R. (1988). M-VAC (methotrexate, vinblastine, doxorubicin and cisplatin) for advanced transitional cell carcinoma of the urothelium.. J Urol.

[OCR_01329] Stewart F. A., Denekamp J., Hirst D. G. (1980). Proliferation kinetics of the mouse bladder after irradiation.. Cell Tissue Kinet.

[OCR_01334] Stewart F. A., Lundbeck F., Oussoren Y., Luts A. (1991). Acute and late radiation damage in mouse bladder: a comparison of urination frequency and cystometry.. Int J Radiat Oncol Biol Phys.

[OCR_01340] Stewart F. A., Michael B. D., Denekamp J. (1978). Late radiation damage in the mouse bladder as measured by increased urination frequency.. Radiat Res.

[OCR_01318] Stewart F. A. (1985). The proliferative and functional response of mouse bladder to treatment with radiation and cyclophosphamide.. Radiother Oncol.

[OCR_01323] Stewart F., Bohlken S., Begg A., Bartelink H. (1986). Renal damage in mice after treatment with cisplatinum alone or in combination with X-irradiation.. Int J Radiat Oncol Biol Phys.

[OCR_01350] von der Maase H. (1986). Experimental studies on interactions of radiation and cancer chemotherapeutic drugs in normal tissues and a solid tumour.. Radiother Oncol.

[OCR_01345] von der Maase H. (1984). Interactions of radiation and adriamycin, bleomycin, mitomycin C or cis-diamminedichloroplatinum II in intestinal crypt cells.. Br J Cancer.

